# A Triad of Congenital Diaphragmatic Hernia, Meckel's Diverticulum, and Heterotopic Pancreas

**DOI:** 10.1155/2014/725945

**Published:** 2014-04-03

**Authors:** Parkash Mandhan, Amer Al Saied, Mansour J. Ali

**Affiliations:** Department of Pediatric Surgery, Hamad General Hospital, Hamad Medical Corporation, P.O. Box 3050, Doha, Qatar

## Abstract

Congenital diaphragmatic hernia is a common developmental anomaly encountered by paediatric surgeons. It is known to be associated with extradiaphragmatic malformations, which include cardiac, renal, genital, and chromosomal abnormalities. Herein, we report a newborn born with concurrent congenital diaphragmatic hernia, Meckel's diverticulum, and heterotopic pancreatic tissue. This is the first case report of such a triad with description of possible mechanisms of the development.

## 1. Introduction

Congenital diaphragmatic hernia (CDH) is relatively a common anomaly that occurs in 2.4 per 10,000 live births [1]. The survival of patients with CDH is dependent on pulmonary hypoplasia and pulmonary vascular abnormality, but association of extradiaphragmatic malformations also plays a vital role in the outcome. The incidence of 1 or more extradiaphragmatic associated anomalies with CDH has been reported as 46% and considered as a major contributory factor in survival of babies born with CDH [2]. Commonly reported extradiaphragmatic associated anomalies with CDH include cardiac (63%), pulmonary sequestration, renal and genital anomalies, neural tube defects, and chromosomal abnormalities [2, 3]. Meckel's diverticulum (MD), a developmental anomaly of omphalomesenteric duct, is also common in children and the reported incidence is around 2% in the general population. Approximately 4% of patients with MD become symptomatic and require surgical excision [4]. Heterotopic pancreas (HtP), that is, the pancreatic tissue lacking anatomical and vascular continuity with the main body of gland, has an incidence of 2–15% in cadaveric studies and 0.5% in the clinical studies [5]. HtP in pediatric age group is not common and may cause a diagnostic dilemma when it leads to complications [5–7].

The association between CDH and MD [8] and also MD associated with HtP has also been reported [5]. However a concurrent association between CDH, MD, and HtP has not been reported previously and we present first case report of such a triad. We have reviewed the possible molecular embryological mechanism of the development of this triad and have also discussed the management.

## 2. Case Report

A 35-week preterm male neonate was born to a 29-year-old healthy mother. Antenatal ultrasound at the 32-week gestation showed left-sided CDH with no additional anomalies noted on scan. After delivery, the baby was intubated and further evaluation confirmed left CDH and no other associated malformations. On the 3rd day of life, after stabilization, he was taken to operating room for repair of his left CDH through transabdominal approach. Operative findings revealed classical posterolateral CDH with herniation of the stomach, small bowel, colon, and spleen into left hemithorax, with no malrotation of the bowel. Further examination revealed a 1 × 1 cm wide base MD over the antimesenteric border of the ileum almost 40 cm from the ileocecal junction. The palpation of MD did not reveal any nodule/mass within it. In addition, there was an isolated 3 × 1 cm yellow, well-circumscribed, lobulated mass over the antimesenteric border of jejunum approximately 15 cm distal to the duodenojejunal junction. Close inspection of this mass was suggestive of an ectopic pancreatic tissue, which was extending from serosa to the lumen of jejunum and had no anatomical and vascular continuity with the main body of the pancreas. The pancreas and hepatobiliary tract were anatomically normal in shape and position.

**Figure 1:**
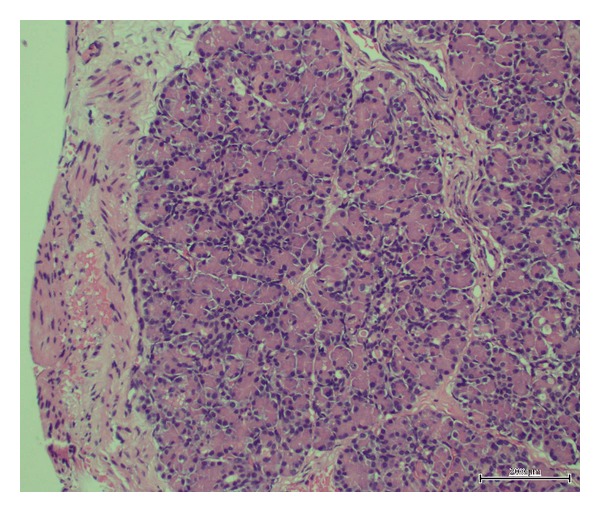
Histology of resected tissue showing high power details of benign pancreatic exocrine acinar tissue (typical of pancreatic heterotopia) (haematoxylin and eosin stain; original magnification, ×200).

The diaphragmatic defect was closed primarily after reducing the contents from left hemithorax. Later the jejunal pancreatic nodule was gently dissected from serosa to mucosa and removed in a single piece. Through enterotomy, jejunum was examined and noted to be healthy with no any more ectopic tissue inside the jejunum. The enterotomy was closed followed by digital examination of MD and it was confirmed to be empty with no contents inside; hence, it was not excised. Patient tolerated surgical procedure very well and his postoperative recovery was smooth. A repeat abdominal ultrasound on the 7th postoperative day was performed, which documented normal pancreas and no other associated anomalies. Histopathological examination of resected specimen showed benign pancreatic exocrine acinar tissue (typical of pancreatic heterotopia) as shown in Figure 1. In a regular followup after 18-months of surgery, he has been free of any complications.

## 3. Discussion

To the best of our knowledge, this is the first case report of concurrent malformations of CDH, MD, and HtP in a neonate.

The true embryogenesis of this triad is unclear, as these malformations are known to occur due to alterations in the morphogenesis of different embryonic structures at various times. The development of diaphragm starts as early as the 4th week of gestation and involves fusion of septum transversum, pleuroperitoneal membranes, esophageal mesentery, and body wall mesoderm [1]. Failure of closure of pleuroperitoneal canals results in visceral herniation of abdominal contents, which affects development of the lung and results in lung hypoplasia. The dorsal and ventral pancreatic buds start to form in the 4th week of gestation and fuse to form the pancreas in the 7th week of gestation. Various theories have been put forward to explain the occurrence of HtP such as the anomalous separation of the developing pancreatic anlagen, with bowel wall penetration and subsequent distant transport, or differentiation of the totipotent endodermal cells in the intestinal tract into pancreatic tissue [9]. MD, an anomaly of omphalomesenteric duct development, occurs due to incomplete obliteration of the omphalomesenteric duct at the 7th week of embryonic development [4].

The developmental processes of diaphragm, gut, and its accessory organs such as pancreas, occur from endodermal and mesodermal embryonic layers and this requires a close interaction between numerous signals such as sonic hedgehog (shh) and their key transcription regulators to transform these layers into distinct sets of precursor cells and specific developed structures [10–12]. Shh, which is expressed from notochord, has been shown to play a pivotal role in formation and differentiation of the gut and its accessory organs [12, 13]. Mandhan et al. observed that shh downregulation results in poor expression of its targets, BMP4 (bone morphogenetic protein), and Hox genes in hindgut development [13]. Kim and Melton observed that permissive signals from notochord are thought to induce pancreatic genes by suppressing shh expression in the dorsal pancreatic epithelium and consider this as one of the ways to develop HtP [14]. Unger et al. studied the role of shh in the pathogenesis of lung hypoplasia in CDH and noted that in pulmonary hypoplasia associated with CDH, the expression of shh is downregulated, which may contribute to the pulmonary abnormality in CDH [11]. In another study of 13 MD specimens, van den Brink and associates found a correlation between certain pathological epithelial differentiation into gastric fundic gland type epithelium and abnormal shh expression [15]. Hence, in our opinion, since shh pathway is known to play a crucial role in the development of these organs at various developmental stages, the downregulation of shh and its targets such as BMP during early embryogenesis may also have a contributory role in the development of this triad of CDH, MD, and HtP in a case like this. However, this proposition would have been validated with additional experimental techniques such as in situ hybridization, which in our case was not performed due to technical limitations.

HtP is usually found in the submucosa or occasionally in subserosa of the upper gastrointestinal tract or within a Meckel's diverticulum, but it has been reported to be in unusual locations such as lungs, gallbladder, spleen, umbilicus, fallopian tubes, lymph nodes, mediastinum, salivary gland, and retroperitoneum [5, 9]. It can occur in multiple locations in the same patient. While patients with HtP are asymptomatic and it is usually an incidental finding during diagnostic procedures, yet depending on the size, depth, and location it may cause a number of complications such as pancreatitis, pseudocyst, gastrointestinal bleeding, small bowel obstruction, intussusception, and transformation to pancreatic adenocarcinoma, which can lead to both diagnostic and management dilemmas. [5–7]. Although there has been a difference of opinion for the resection of HtP when it is detected intraoperatively, currently many authors support aggressive resection of resectable HtP in a view of a number of reported serious complications [5–7, 9]. In our case, HtP was large and extending from serosa to the mucosa of bowel; hence we proceeded for the resection of HtP to avoid the risk of future complications. Pathologically HtP has been subdivided into four subtypes; type I heterotopia consists of typical pancreatic tissue; type II shows pancreatic ducts only, type III consists of acinar tissue only; and type IV has islet cells only [16, 17]. In our case, it was of type III as only pancreatic exocrine acinar tissue was noted in the resected tissue (Figure 1).

The management of incidentally found MD remains also controversial [18–20]. The risk for future complications of a nonresected MD must be weighed against the risk of complications for resected MD in order to justify a prophylactic resection. The reported morbidity rates after removal of incidentally found asymptomatic MD are much lower than those after resection of symptomatic MD [19, 20]; hence, the decision lies over the operating surgeon. In our case, we could not justify the prophylactic removal of asymptomatic MD due to multiple factors such as small size of MD, no palpable tissue inside the MD, a previous enterotomy in proximal bowel to resect HtP, and associated other surgical procedures for CDH. We do plan to followup this patient throughout childhood in order to identify any complication secondary to MD.

## 4. Conclusion

In summary, the actual cause of concurrent CDH, MD, and HtP in our patient is not known but we consider that the disruption of common shh signaling pathway for the development of these structures during early embryonic life may have been a lead factor to this triad. In clinical practice, it is important to remember that CDH, MD, or HtP might accompany each other and need appropriate attention to reduce the risk of future complications.
